# Proteomics and Metabolomics Profiles of Unvaccinated Nonagenarian Patients with Severe SARS-CoV‑2 Infection

**DOI:** 10.1021/acs.jproteome.5c00251

**Published:** 2025-09-25

**Authors:** Mauricio Quiñones-Vega, Patricia Sosa-Acosta, Jéssica de Siqueira Guedes, Natália Pinto de Almeida, Mateus V de Castro, Moníze V. R. Silva, Luiz P. Dell’Aquila, Álvaro Razuk-Filho, Pedro B. Batista-Júnior, Mayana Zatz, Fábio César Sousa Nogueira, Gilberto Barbosa Domont

**Affiliations:** † Proteomics Unit, Department of Biochemistry, Institute of Chemistry, 28125 Federal University of Rio de Janeiro, Rio de Janeiro, Rio de Janeiro 21941-909, Brazil; ‡ Laboratory of Proteomics, LADETEC, Institute of Chemistry, Federal University of Rio de Janeiro, Rio de Janeiro, Rio de Janeiro 21941-598, Brazil; § Precision Medicine Research Center, Institute of Biophysics Carlos Chagas Filho, Federal University of Rio de Janeiro, Rio de Janeiro 21941-902, Brazil; ∥ Human Genome and Stem Cell Research Center, 28133 University of São Paulo, São Paulo, São Paulo 05508-000, Brazil; ⊥ Prevent Senior Institute, São Paulo, São Paulo 04547-100, Brazil

**Keywords:** COVID-19, biomarkers, nonagenarians, plasma, proteomics and metabolomics

## Abstract

The mortality rate of COVID-19 increases significantly in patients over the age of 90, although some elderly people in this category have experienced mild disease or have been asymptomatic. In this context, we aim to analyze the plasma proteomic and metabolomic profiles of unvaccinated nonagenarian patients who had severe manifestations of COVID-19 and either recovered or died and compare them with noninfected control subjects. Compared with healthy individuals, nonsurviving patients showed a reduced abundance of specific lipid-related proteins and metabolites, including APOH, APOC1, LCAT, 7-α-25-dihydroxycholesterol, 7-dehydrocholesterol, and phosphatidylethanolamine, which may serve as indicative markers of severity. Acute phase response, complement activation, and sphingolipids and phospholipids remain altered in recovered patients, indicating possible persistent effects of COVID-19. This study employs a multiomics approach to unveil key immune alterations in unvaccinated nonagenarians, shedding light on molecular signatures that may serve as predictive markers for disease severity and recovery in the elderly population.

## Graphical abstract

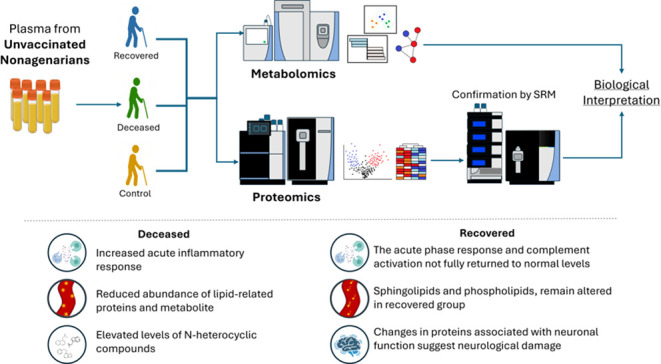

## Introduction

The outbreak of the new coronavirus, SARS-CoV-2, in December 2019 resulted in more than 700 million confirmed cases of infection and over 7 million deaths globally.^1^ Although the disease often manifests as a mild respiratory tract infection, approximately 20% of patients remain asymptomatic. However, severe symptoms, such as pneumonia, acute respiratory distress syndrome (ARDS), and significant immune dysregulation, can develop in some individuals. It remains unclear whether disease severity is primarily driven by direct viral effects or by the excessive immune response known as the cytokine storm.^2^ Many studies describe different clinical features that increase the probability of severe outcomes.^3–5^ For example, sex, obesity, diabetes, cardiovascular complications, and cancer are among the most common risk factors for the severity of the disease.^6,7^ One of the most described risk factors is age. The mortality rate drastically increases in patients older than 60 years.^5^ Li et al. (2020) investigated the incidence and mortality rate in 5,319 infected patients and 76 fatal cases, reporting that despite the fact that COVID-19 can occur in all age groups, the estimated fatal probability is 0.51 for people older than 90 years.^8^ Also, Levin et al. found an exponential relationship between infection fatality rate and age. This rate increases from 0.002% at age 10 to 15% at age 85.^9^ This phenomenon is probably due to immunosenescence among the elderly, which contributes to altered activation of the innate immune response that leads to cytokine release syndrome, tissue damage, and uncontrolled viral replication with fatal clinical consequences.^10^

The comprehensive study of different manifestations of the disease in senior patients contributes to the identification of intrinsic and individual factors of the immune response that may confer protection or susceptibility to SARS-CoV-2 infection. An ideal sample for this type of study is plasma because it represents the individual’s status and phenotype and is routinely collected in most clinical studies. This sample contains both proteins and metabolites that can describe the physiological conditions and the specific changes that typically monitor the onset and progression of the disease. MS-based proteome and metabolome analysis of plasma and serum are unbiased and, in principle, an ideal technology to characterize the entire disease response system.^11^ Although body fluids are very challenging, multiomics-based technologies have been extensively employed to determine the SARS-CoV-2 infection mechanism and COVID-19 pathogenesis.^11–13^ Chen et al. (2020) combined transcriptomics, proteomics, and metabolomics to analyze the plasma of 16 severe and 50 mild COVID-19 patients and found chronic activation of neutrophils, IFN-I signaling, and a high level of inflammatory cytokines.^14^ A proteomic and metabolomic study of COVID-19 patients with 6-month survivors revealed that abnormalities persist after hospital discharge, especially in terms of the extracellular matrix, immune response, and hemostasis pathways.^15^ Krishnan et al. (2021) used plasma proteomics and metabolomics of COVID-19 patients to understand the role of key metabolic pathways underlying SARS-CoV-2 infection, showing significantly raised levels of cytokines and chemokines, such as IL-6.^16^ Therefore, multiomics approaches are invaluable tools to characterize plasma profiles in severe COVID-19 patients, including infection and recovery of elderly individuals, who are at a higher risk in the context of the disease.

In this study, we used proteomic and metabolomic approaches to identify the main differences between nonagenarians’ COVID-19 patients who either survived or did not survive, compared to noninfected control subjects of similar age. Our study included patients over 90 years old before the vaccination period, making it of significant importance since this type of sample is rare, scarce, and difficult to collect nowadays. The plasma proteome and metabolome can provide a comprehensive view of molecular changes, especially in patients who exhibit distinct immune responses. The aim is to enhance our understanding of alterations in protein and metabolite profiles throughout the infection and recovery stages, as well as potential sequelae experienced by elderly patients.

## Methods

### Study Cohort

Plasma samples from nonagenarians infected with SARS-CoV-2 (Table 1) were obtained from the Human Genome and Stem Cell Research Center (HUG-CELL) at the University of São Paulo (USP), Brazil. We classified the samples into three categories: control subjects who tested negative in both PCR and serological tests for SARS-CoV-2 (Group C, *n* = 3); patients who were severely infected and did not survive (Group D, *n* = 3); and patients who recovered after a severe infection (Group R, *n* = 3). The severity of COVID-19 was classified according to the clinical spectrum outlined in the World Health Organization’s updated guidelines for COVID-19 treatment.^17^ These samples are part of a larger cohort known as the “Resilient Brazilian Super-Elderly”.^18^

**Table 1:** Cohort under Analysis

Serum samples	COVID-19 diagnosis^a^	COVID-19 severity	Sample collection	Outcome	Sample group	Sex^c^	Age
01, 02, 03	Positive	Severe	4–19 days after infection^b^	Deceased	D.	F, M, M	95, 95, 92
04, 05, 06	Positive	Severe	30–90 days after infection	Survival	R.	F, M, F	93, 94, 91
07, 08, 09	Negative	-	30–90 days after exposure	-	C.	F, F, M	100, 97,101

a Based on the PCR and serological tests for SARS-CoV-2.

b During hospitalization in the ICU.

c Where F stands for female and M stands for male.

All patients with severe COVID-19 were admitted to an intensive care unit (ICU), exhibiting symptoms of dyspnea, hypoxemia, and pulmonary impairment. Table S1 summarizes detailed clinical information about the disease and comorbidities. Peripheral blood samples were collected using Vacutainer tubes containing ethylenediaminetetraacetic acid (EDTA) (BD Biosciences, Catalog #360057) before the availability of COVID-19 vaccines in Brazil and before the appearance of new SARS-CoV-2 variants (between June and October 2020). Plasma was obtained by centrifugation at 2,000 g for 10 min at room temperature within 30 min after venipuncture. The samples were stored immediately at −80 °C until analysis.

Confirmation of the SARS-CoV-2 infection was initially determined by PCR when individuals began exhibiting symptoms. Subsequently, plasma collected from these individuals underwent serological analysis (IgA, IgM, and IgG anti-SARS-CoV-2) to further confirm the infection. A PCR test was performed at the time of exposure, followed by serological testing to definitively rule out infection for those exposed but not infected. Additionally, these noninfected individuals displayed no flu-like or COVID-19-related symptoms up until the time of sample collection.

### Sample Processing for Proteomics

For the proteomic analysis, the volume equivalent to 100 μg of proteins was diluted 20-fold with 5% SDS/50 mM TEAB at pH 8.5. The samples were reduced with DTT for 1 h at 30 °C and alkylated with 40 mM IAA for 45 min at room temperature in darkness. After acidifying the mixture with phosphoric acid to a final concentration of 1.2%, 350 μL of 90% methanol/100 mM TEAB (binding buffer) was added. The samples were then placed on an S-Trap MIDI spin column (Protifi, USA) and centrifuged at 4000 g for 30 s. Binding buffer (450 μL) was used three times to remove the detergent. Digestion was performed using trypsin (Promega) at a ratio of 1:50 (μg trypsin: μg protein) in a final volume of 125 μL of 50 mM TEAB at pH 8.5 for 18 h. The peptides were eluted stepwise with four solutions: 80 μL of 50 mM TEAB at pH 8.5, 80 μL of 0.1% formic acid (FA), 40 μL of 50% acetonitrile (ACN)/0.1% FA, and 40 μL of 70% ACN/0.1% FA, followed by centrifugation at 4000 g for 60 s. The samples were dried using SpeedVac (SAVANT, Thermo Fisher Scientific) and stored at −30 °C.

### Depletion of Plasma Proteins

In parallel, we depleted a pool of plasma samples of the 14 most abundant proteins using the Multiple Affinity Removal Column Human 14 (4.6 × 100 mm, Agilent) according to the manufacturer’s instructions. The depletion procedure was performed on a Dionex UltiMate 3000 UHPLC instrument (Thermo Scientific, USA). The unbound fraction (depleted plasma) was then processed and digested in S-Trap spin columns as mentioned previously.

### Protein and Peptide Quantification

Protein and peptide concentrations were verified using the Qubit Protein Assay (Invitrogen, Thermo Fisher Scientific, USA), following the manufacturer’s instructions.

### Sample Processing for Metabolomics

Metabolites contained in 100 μL of plasma samples were extracted with cold methanol (1:6, v/v) spiked with deuterated testosterone (50 nM of Testosterone-D3, LGC Standards, London, England) as an internal standard and incubated for 30 min at −30 °C. The samples were then centrifuged at 14,000 g for 15 min at 4 °C. The supernatant was collected and dried using SpeedVac (Thermo Scientific). Dried metabolites were stored at −80 °C until analysis.

Ten microliters from each sample was pooled and used as quality control (QC) samples to monitor LC-MS performance. Blanks were included to detect interferences. All samples were processed with cold methanol containing deuterated testosterone to assess system performance and data robustness. To ensure reproducibility, QC and blank samples were analyzed after every six and 12 patient samples, respectively. Patient samples were processed and analyzed in random order to prevent batch effects.

### Mass Spectrometry Analysis for Proteomics (nLC-MS/MS)

We resuspended the tryptic peptide mixtures in 0.1% formic acid and applied two μg of peptides in duplicate to an Easy-nLC 1000 system (Thermo Fisher Scientific) coupled online to a Q-Exactive Plus mass spectrometer (Thermo Fisher Scientific). We loaded the peptides into a trap column, Acclaim PepMap 100 C18 HPLC Column, with 20 mm (3 μm spheres, 75 μm ID, Thermo Fisher Scientific, USA). Then, we fractionated them in an EASY-Spray column, 50 cm × 75 μm ID, PepMap RSLC C18, 2 μm (Thermo Fisher Scientific, USA). The mobile phases were 5% ACN/0.1% formic acid (solvent A) and 95% ACN/0.1% formic acid (solvent B). We separated the peptide mixture using a gradient: 5–30% B for 112 min, 30–38% B for 10 min, 38–58% B in 10 min, 58–93% B in 5 min, and 93% B for 23 min. The flow rate was 300 nL/min, and the temperature was 60 °C.

The data-dependent acquisition (DDA) method selects the 20 most intense peptides for fragmentation by high-energy collision dissociation (HCD), using a collision energy of 30 (NCE). For the full scan analysis (MS1), we set the Orbitrap resolution to 70,000 (*m*/*z* 200), the AGC (automatic gain control) to 1 × 10^5^, and the maximum injection time to 50 ms. In the case of obtaining the fragmentation spectra (MS2), an Orbitrap resolution of 17,500 (*m*/*z* 200), an AGC of 1 × 10^5^, a maximum injection time of 100 ms, and an isolation window of *m*/*z* 2 were used. The dynamic exclusion time was 45 s.

### Mass Spectrometry Analysis for Metabolomics (LC-MS/MS)

We analyzed the metabolites (10 μL) using a Q-Exactive Plus mass spectrometer (Thermo Scientific) coupled to a UHPLC Ultimate 3000 system (Thermo Fisher Scientific). Metabolites were separated on a reverse-phase Zorbax Extend C18 column (2.1 × 150 mm, 1.8 μm; Agilent Technologies) with an oven temperature of 40 °C. The mobile phases employed were 0.1% formic acid/5 mM ammonium formate, pH 3.0 (solution A), and 0.1% formic acid/methanol (solution B). Metabolites were separated using a gradient starting at 5% B for 12 s, 5–10% B for 18 s, 10–25% B for 30 s, 25–90% B for 6 min, 90–100% B for 4 min, and 100% B for 2 min at a flow rate of 0.40 mL/min.

The mass spectrometer was operated in full MS/dd-MS2 mode with an acquisition range of 70–800 *m*/*z* for positive and negative ionization modes (PI and NI). The spray voltage was set to 3.9 and 2.9 kV for PI and NI, respectively, with a capillary temperature of 380 °C for both modes. The MS1 was configured with a resolution of 70,000 resolution, an AGC target of 1 × 10^6^, and a maximum IT of 100 ms. MS2 was acquired at a resolution of 17,500, an AGC target of 1E^5^, a maximum IT of 50 ms, and a loop count of 10. Dynamic exclusion was set to 30 sec, with an isolation window of *m*/*z* 2.0. Metabolites were fragmented in an HCD cell with 30% NCE.

### Selected Reaction Monitoring Methodology

After the characterization of the total proteome and evaluation of the results, the altered proteins were confirmed by the selected reaction monitoring (SRM) approach. The digested plasma proteins (4 μg) were analyzed on a UHPLC Ultimate 3000 (Thermo Fisher Scientific) coupled to a TSQ Quantiva mass spectrometer (Thermo Fisher Scientific). Tryptic peptides were separated on an EclipsePlus C18 column (2.1 × 150 mm, 1.8 μm; Agilent Technologies) with 0.1% formic acid as solvent A and ACN/0.1% formic acid as solvent B, using the following gradient: 5–35% B in 11 min, 35–90% B in 2 minutes, and held at 90% B for 1 min. The spray voltage at the ESI source was set to 3.5 kV in positive mode, and the ion transfer and vaporizer temperatures were 350 and 400 °C, respectively. The resolution for Q1 and Q3 was 0.7 for both analyzers. Optimal collision energy was predicted by Skyline 21.2 software (MacCoss Lab) according to a linear equation for a TSQ Quantiva mass spectrometer.

### Data Processing for Untargeted Proteomics

The analysis of the data obtained was conducted using Proteome Discoverer 2.4 (PD 2.4) software (Thermo Fisher Scientific). For the processing of raw data, the MSPepSearch node was used to search a spectral library available at https://www.proteometools.org/(316671 spectra) with 10 ppm as precursor mass tolerance and 0.6 Da as fragment mass tolerance. After validation with the Percolator node, the unidentified MS2 spectra were filtered and analyzed using the SequestHT algorithm. The search was carried out against the *Homo sapiens* database (42,264 reviewed canonical and isoform sequences) deposited at UniProt (https://www.uniprot.org/). Two missed cleavages and semitryptic peptides were set as search parameters. Fixed modifications included carbamidomethylation (C), and for variable modifications, methionine oxidation and acetylation of the protein N-terminal were accepted. The mass tolerance of the precursor was 10 ppm, and for the ion fragment, it was 0.1 Da. For protein and peptide validation, the false discovery rate (FDR) was less than 1% at the peptide level and 5% at the protein level, and proteins were grouped into master proteins using the maximum parsimony principle. Protein quantification was accomplished using the extracted ion chromatogram (XIC) approach, performing relative quantification according to the peak area of the three most abundant distinct peptides of each protein in the workflow node “Precursor Ions Area Detector” in PD 2.4. Match between runs was used with a retention time shift of 5 min.

### Data Processing for Targeted Proteomics

Skyline 21.2 software (MacCoss Lab) was used to define 2–3 unique peptides (6–25 aa) per protein of interest. We excluded peptides containing methionine and glycosylation sites, and all cysteines were considered carbamidomethylated. Precursors with two charges were considered, along with the 6–3 most intense “y” and “b” ions for transitions, based on a spectral library obtained from the DDA analysis. The normalized area of each peak was determined by spiking the heavy-labeled peptide “R­(+10)­QYFWIAWYK­(+8)­LANSK” as an external standard.

### Data Processing for Untargeted Metabolomics

Compound Discoverer software (version 3.2, Thermo Scientific) was employed for raw data refinement, metabolite identification, and quantification using the following workflow: Untargeted Metabolomics with Statistics, Detect Unknowns with an ID using Online Databases, and mzLogic. The maximum retention time shift for the Alignment Retention Time Node was set to 3 min. Metabolite identification was performed through data comparison with the HMDB, LIPIDMAPS, NIST, KEGG, and mzCloud databases. Metabolite identification with accurate precursor mass and MS2 spectra, confirmed using external libraries, delivers a level 2 identification.^19^ All nodes with mass tolerance parameters were set to a value of 5 ppm. Criteria for feature filtration included ensuring that the name was not blank, the normalization area had any value, and the name did not contain “similar” (clusters were excluded); the feature was identified with MS2, had a full match in any annotation source, and exhibited an RSD lower than 60% before QC file correction. Xenobiotics were removed manually by utilizing the HMDB and CHEBI xenobiotic databases.

### Proteomics Statistical and Functional Analysis

The list of master proteins was imported into Perseus software (version 1.6.15.0) for data analysis. Only proteins detected in at least two biological replicates in each group were used for the statistical analysis. The average protein abundance was converted to log2 and normalized by subtracting the median of the sample distribution. Three normalized fold-change (FC) groups were created for the analysis of differentially abundant proteins: D vs C, D vs R, and R vs C. A Student *t* test was performed, considering proteins significant if their *p*-values for FC were lower than 0.05. Enrichment analysis of GO annotations was performed using the ClusterProfiler R package (version 4.6.2).^20^

### Metabolomics Statistical and Functional Analysis

Statistical and functional analyses were performed in MetaboAnalyst (version 5.0).^21^ Perseus software (version 1.6.15.0) was used for statistical analysis, including Student’s *t* test, ANOVA, and heatmap generation, considering a *p-*value < 0.05. Student’s *t* test was employed for the following comparisons: D vs C, D vs R, and R vs C. PatternHunter analysis was performed using Pearson’s for distance measure calculations and considering metabolites with the C-R-D pattern. Boxplot graphics were created using the ggplot R package.

### Interaction Networks

Metabolite–metabolite and metabolite–protein interaction networks were analyzed using the MetaboAnalyst platform (version 5.0).^21^ Statistically significant metabolites and proteins for the D vs C comparison were included. Only metabolites with HMDB codes were considered.

## Results

The main objective of this study is to characterize the proteomic and metabolomic alterations in nonagenarian subjects infected with COVID-19 who either did not survive or successfully recovered, aiming to identify markers of susceptibility or protection against the disease. To achieve this, we investigated two biological groups: deceased nonagenarian patients (Group D) and nonagenarian patients who recovered from COVID-19 (Group R), both experiencing severe manifestations of the disease. Additionally, a control group (Group C) of healthy nonagenarian subjects not infected by SARS-CoV-2, despite exposure, was included for comparison (Figure 1A). Untargeted analyses were used to obtain proteomic and metabolomic plasma profiles. Using a targeted approach, we also confirmed the presence of a set of altered proteins in these subjects.

**Figure 1:**
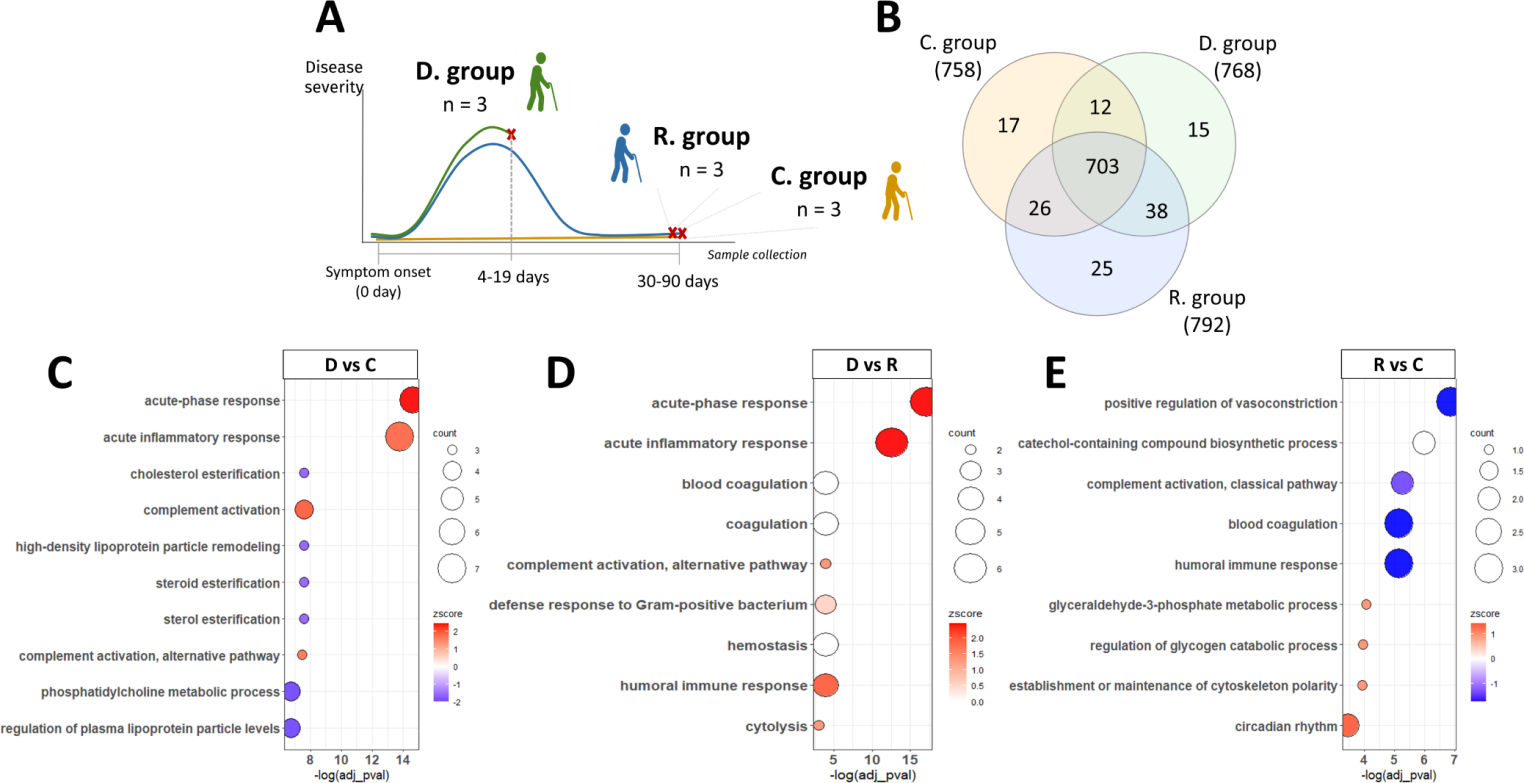
Label-free quantitative proteomic characterization of the control (C), recovered (R), and deceased (D) patients over 90 years. (A) Overview of the sample collection carried out in this study. (B) Venn diagram representing the total proteins identified in each group: C, D, and R. (C–E) Enriched GO terms found in differentially abundant proteins in D vs C; D vs R, and R vs C, respectively. The Z-score represents up- or downregulation of GO terms depending on the fold change of the genes. The area of the displayed circles is proportional to the number of genes assigned to the term.

The subjects over 90 years of age and the unvaccinated condition limited the samples included in this study. However, despite the low number of subjects, our data highlight significant insights into global proteomics and metabolomics alterations in nonagenarian patients during COVID-19 infection. These findings demonstrate key differences when compared to a similar control group, showing the impact of severe infection on lipid metabolism and inflammatory response, alongside markers associated with disease severity and recovery, thereby contributing to the understanding of SARS-CoV-2’s effects in this unique and vulnerable population. Importantly, the findings were confirmed using two complementary approaches—proteomics and metabolomics—aligned with observations reported in prior research, reinforcing their relevance and robustness.

### Proteomic Alterations in Severe and Recovered Nonagenarian COVID-19 Patients

Using a label-free quantitative proteomics approach, we identified 836 proteins from all nine plasma samples, 703 of which were common to all samples (Table S2). In the control, deceased, and recovered groups, we found 17, 15, and 25 proteins exclusively, respectively (Figure 1B). The Pearson correlation values were all greater than 0.9 between all proteomics profiles, and the data fit a normal distribution.

To identify the differentially expressed proteins between the three groups, we performed an ANOVA and three Student’s *t* tests. We applied a multiple sample test ANOVA (*p*-value <0.05) (Table S3) to identify proteins that were differentially abundant across all groups. This test reported 38 proteins (Table 2) mainly associated with the acute-phase response, complement activation, and regulation of plasma lipoprotein particles.

**Table 2:** Proteins Differentially Expressed between the Three Groups (D, R, and C) According to Multiple Sample Test ANOVA (*p*-Value <0.05)

Accession	Gene name	–Log ANOVA *p*-value	D vs C fold change	–Log *p*-value D vs C	D vs R fold change	–Log *p*-value D vs R	R vs C fold change	–Log *p*-value R vs C
P0DJI8	SAA1	3.63	8.00	3.97	7.09	2.37	-	-
P01011	SERPINA3	2.77	1.38	2.24	1.36	1.60	-	-
P51512	MMP16	2.55	–0.77	1.53	––1.34	2.80	-	-
Q8IV33	KIAA0825	2.27	–1.87	2.08	–1.74	2.07	-	-
P01859	IGHG2	1.94	–1.42	1.61	-	-	-	-
P09172	DBH	1.92	-	-	-	-	–4.63	2.22
Q9UGM5	FETUB	1.89	-	-	-	-	-	-
P02748	C9	1.88	-	-	0.67	2.07	-	-
O75015	FCGR3B	1.82	4.82	2.31	-	-	5.54	1.83
P34096	RNASE4	1.82	0.66	1.36	1.10	1.80	-	-
P00488	F13A1	1.82	-	-	-	-	-	-
P20711	DDC	1.80	-	-	-	-	-	-
A0A075B6K0	IGLV3–16	1.76	1.95	2.53	-	-	-	-
P43652	AFM	1.63	–1.07	2.15	–0.72	1.52	-	-
P80108	GPLD1	1.63	–0.91	1.37	–0.63	0.96	-	-
P62136	PPP1CA	1.61	-	-	-	-	-	-
P43251	BTD	1.60	-	-	–1.02	1.49	-	-
Q9UK55	SERPINA10	1.59	-	-	-	-	-	-
A0A0C4DH33	IGHV1-24	1.59	3.76	1.63	-	-	2.94	1.44
Q16853	AOC3	1.58	-	-	-	-	-	-
P00918	CA2	1.58	1.52	1.62	-	-	-	-
P07358	C8B	1.53	-	-	0.94	1.52	-	-
P52566	ARHGDIB	1.52	-	-	-	-	-	-
P02763	ORM1	1.50	1.24	1.48	-	-	-	-
Q9HDC9	APMAP	1.49	-	-	-	-	–2.34	1.81
Q9BXR6	CFHR5	1.48	2.26	1.68	-	-	-	-
P33151	CDH5	1.48	-	-	-	-	-	-
P02741	CRP	1.48	7.23	2.42	-	-	-	-
P03952	KLKB1	1.45	-	-	-	-	-	-
P04278	SHBG	1.45	-	-	–0.75	1.38	-	-
P0DJI9	SAA2	1.43	8.38	1.47	7.06	1.31	-	-
P04180	LCAT	1.43	-	-	–0.69	1.43	-	-
P01009	SERPINA1	1.40	-	-	-	-	-	-
P02647	APOA1	1.40	-	-	-	-	-	-
P02654	APOC1	1.37	–0.84	1.45	-	-	-	-
Q8NFU5	IPMK	1.36	-	-	-	-	-	-
Q6UXN8	CLEC9A	1.34	–0.89	1.68	-	-	-	-
Q16270	IGFBP7	1.32	-	-	-	-	-	-

We performed three Student’s *t* tests (Table S4) to determine which proteins are differentially expressed among the three groups. To find circulating proteins associated with COVID-19 mortality, we compared the group of infected nonsurvivor patients to the control group (D vs C) and the recovered group (D vs R). The D vs C comparison reported 43 differentially expressed proteins, while D vs R resulted in 24 proteins. The enrichment analysis showed, in both cases, that the main biological processes upregulated were the acute-phase response and the acute inflammatory response (Figure 1C,D respectively). To find proteins that remain differentially expressed during the recovery stage of the infection, we analyzed the R group relative to the C group (R vs C) resulting in 9 differentially expressed proteins. In this case, the main annotations enriched are related to the regulation of vasoconstriction and extrinsic apoptotic pathways (Figure 1E). We found 25 common proteins between the ANOVA and *t* test analyses (Table 2). Proteins like CRP, SERPINA3, SAA1, SAA2, and immunoglobulins were previously related to SARS-CoV-2 infection in the literature,^22–24^ and their abundance increases during the infection and returns to their normal status after 3–4 weeks postinfection. However, we found, by using ANOVA and *t* test, four proteins (DBH, IGHV1-24, FCGR3B, APMAP) that remain altered after 20–30 days and could be related to infection sequelae. Hierarchical clustering was performed on all 78 proteins that showed significant changes in all statistical tests (Figure 2). Samples from the same group were clustered, except for the recovered group, which displayed an intermediate behavior. Three clusters of proteins were defined: one with proteins that decreased in the D group relative to the R and C groups (cluster 1), another with proteins that increased in the D group relative to other groups (cluster 2), and a third cluster with proteins that decreased in the R group (cluster 3) (Figure 2B). The GO biological processes enriched in each cluster showed regulation of lipoprotein particles mainly associated with cluster 1, an acute-phase inflammatory response more represented in cluster 2, and a humoral immune response enriched in cluster 3 (Figure 2C).

**Figure 2:**
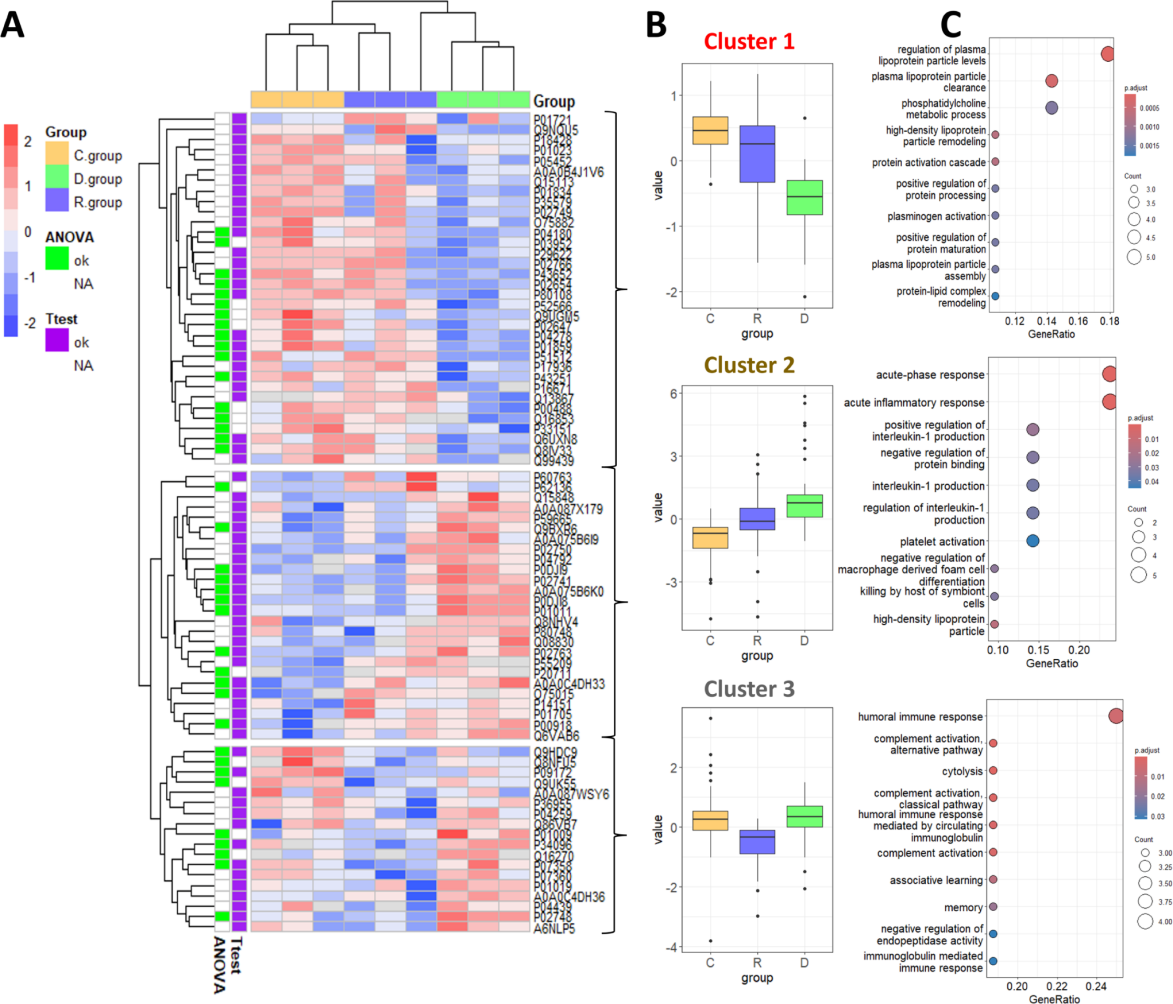
Analysis of the proteins differentially expressed in D, R, and C groups according to ANOVA and the *t* test. (A) Hierarchical clustering by Euclidean distance of all statistically significant proteins (*N* = 78). The scale bar represents protein abundances normalized for all samples. (B) Boxplots representing the normalized abundance of proteins found in each cluster. (C) GO terms enriched in each cluster. The area of the displayed circles is proportional to the number of genes assigned to the term.

### Targeted Analysis of Biologically Relevant Proteins

To further confirm our results, we created a list of 62 proteins that met the requirements for the correct detection of peptides, from the 78 significant proteins identified in the ANOVA and the Student’s *t* tests. For this, we optimized a selected reaction monitoring (SRM) method to quantify these proteins. In total, we selected 124 precursors with 3–5 fragment ions per precursor, reaching 630 selected transitions. The relative quantification was achieved by adding a labeled nonhuman peptide as a global standard. We also compared each group, as in the untargeted analysis, to identify differentially abundant proteins. We confirmed that 28 proteins were statistically different in the D vs C comparison, from which 7 and 11 were upregulated and downregulated, respectively, in both the untargeted and targeted approaches (Figure 1A). In the D vs C contrast, we found 11 statistically significant proteins, with two and one proteins upregulated and downregulated, respectively, in both approaches (Figure 1B). In the R vs C analysis, we found only one protein differentially abundant (A2MG_HUMAN) (Figure 1C).

### Metabolomic Disruptions Associated with COVID-19 Mortality and Recovery

In addition to proteomics, we developed an untargeted metabolomics analysis, identifying 408 metabolites while considering both positive and negative ionization (Table S5). The most abundant metabolites were lipids and lipid-like molecules, specifically fatty acid esters, fatty acids and conjugates, and ceramides. The second most abundant metabolite subclass consisted of amino acids and peptides. PCA analysis separated the three biological groups under study, with a 44.7% variation in the first component (Figure 3A). The D group was visibly different from the C and R groups, confirming the metabolic disruption characteristic of severely infected patients. On the other hand, the R group was close to the C group, showing almost complete recovery in these patients.

**Figure 3:**
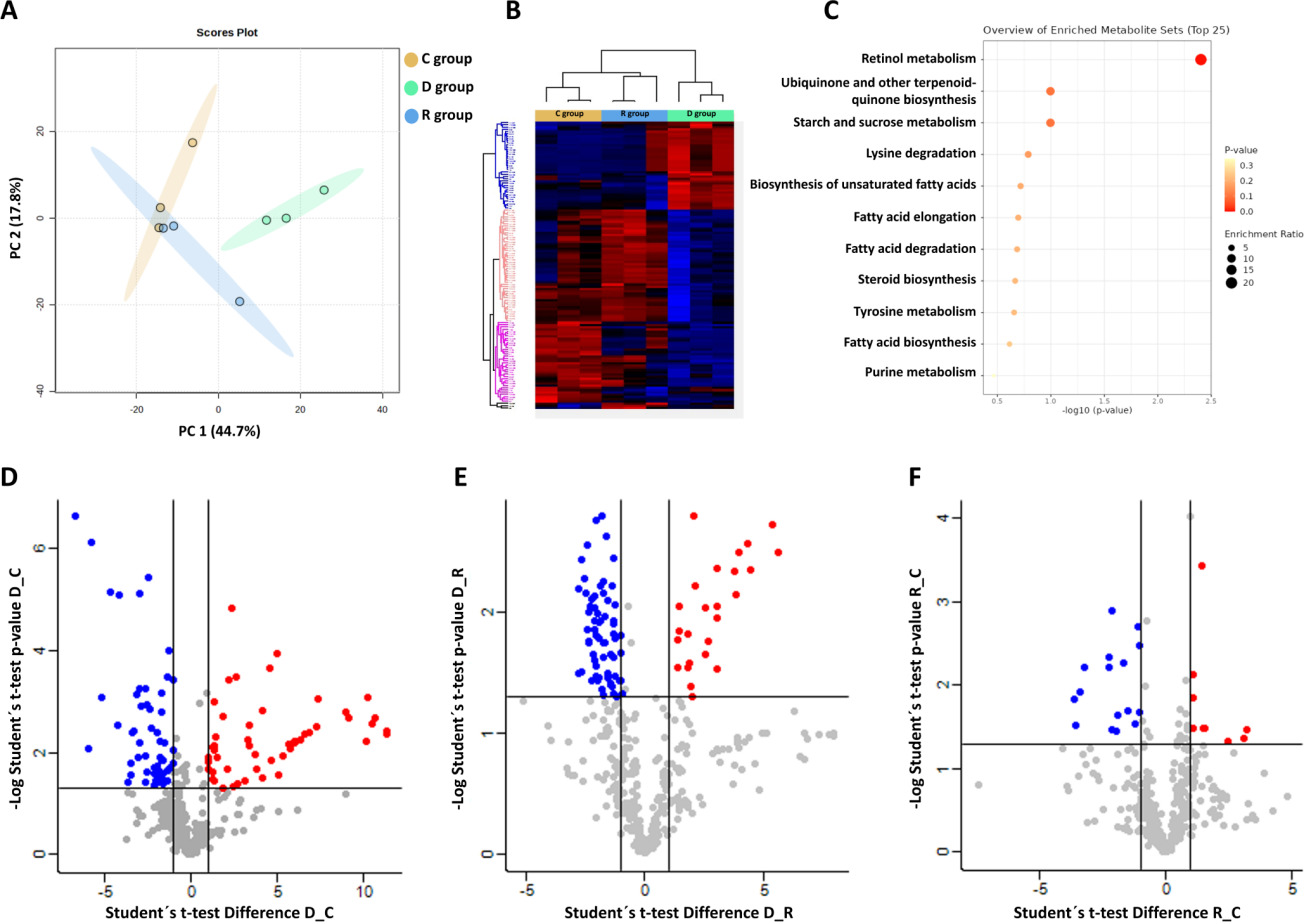
(A) Principal component analysis (PCA) for the three biological groups studied (C, D, and R). (B) Heatmap corresponding to the 128 statistically significant metabolites in the ANOVA test with *p*-value <0.05. (C) Enrichment of metabolic pathways altered throughout C, R, and D groups. (D–F) Volcano plots for (D) D vs C, (E) D vs R, and (F) R vs C.

For metabolomics statistical analysis, the ANOVA and Student’s *t* test were performed considering a *p*-value <0.05 (Tables S6 and S7). In the ANOVA analysis, we identified 128 statistically significant metabolites related to retinol metabolism, ubiquinone biosynthesis, and starch and sucrose metabolism (Figure 3B,C). Most of the metabolites in the infected group exhibited reduced abundance compared to the recovered or control groups. The Student’s*t*test showed 131, 92, and 39 statistically significant metabolites for D vs C, D vs R, and R vs C comparisons, respectively (Figure 3D–F). Among them, 11 metabolites were statistically significant for the three comparisons (Table 3).

**Table 3:** Statistically Significant Metabolites in D vs C, D vs R, and R vs C^a^

Name	D. vs C. fold change	D. vs R. fold change	R. vs C. fold change
2-(cyclohexylmethylidene)-1,2,3,4-tetrahydronaphthalen-1-one	–0.91	–2.03	1.12
lyso-paf c-18	–1.63	–2.48	0.84
19-norandrostenedione	–0.98	–1.82	0.83
1-(4-methyl-2-pyridyl)pyrrolidine-2,5-dione	3.37	2.57	0.80
1-hexaInfyllysophosphatidylcholine	–1.77	–2.36	0.59
(1s,3r,5z,7e,9xi)-18-{[3-(3-hydroxy-3-pentanyl)benzyl]oxy}-20-methyl-9,10-secopregna-5,7,10-triene-1,3-diol	–1.67	–2.12	0.45
juniperic acid	–1.67	–1.03	–0.64
methyl 2-acetamidobenzoate	1.05	2.06	–1.01
ganglioside GM3 (d18:1/24:1(15z))	–2.97	–1.32	–1.65
nonadecanone	–2.90	–0.97	–1.93
(7z,19r,31r)-22,25,28-trihydroxy-22,28-dioxido-16,34-dioxo-31-(palmitoyloxy)-17,21,23,27,29,33-hexaoxa-22lambda ∼ 5∼,28lambda ∼ 5∼-diphosphanonatetracont-7-en-19-yl (5z,8z,11z,14z)-5,8,11,14-icosatetraeno ate	–4.15	–1.89	–2.26

a Comparisons, considering *p*-value <0.05.

The D group exhibited an elevation in nitrogen compounds (i.e., N-heterocyclic compounds) compared to the C group, encompassing saturated and unsaturated cyclic compounds, amides, and amines, all displaying a fold change higher than two. Conversely, the D group was characterized by a decreased abundance of phospholipids, sphingolipids, fatty acids, and cholesterol- and retinol-related metabolites. The disruption of amino acid metabolism was observed, although its activation or inhibition remained unclear. A comparison between the infected group and the recovered patients (D vs R) revealed negative alterations in specific metabolite subclasses, including phospholipids, sphingolipids, fatty acids, and retinol-related metabolites. Ceramides were found to be exclusively down-abundant in this comparison. Among the nitrogen compounds, only the amide derivatives exhibited a decrease in abundance. Some metabolites were not recovered after COVID-19 infection (R vs C), encompassing amino acids and analogues, lipids, catecholamines, and fatty acids. These persistent alterations shed light on the long-term metabolic consequences of COVID-19.

The PatternHunter analysis identified metabolites that increase or decrease according to the C-R-D pattern (Figure 4A). Notably, six nitrogen compounds (Figure 4B) displayed ascending behavior during the C-R-D pattern, with their levels doubling or more during infection, highlighting the relevance of nitrogen metabolism in SARS-CoV-2 infection. Intriguingly, oleoyl ethanolamide and *d*-pyrrolidine-2,5-dione alterations persisted without recovery, remaining elevated in both D vs C and R vs C comparisons. Conversely, suberic acid and *d*-pyran-2-one were significantly impacted by infection, experiencing a 23- and 31-fold increase compared to the C group; yet, this alteration was fully restored in the R group.

**Figure 4:**
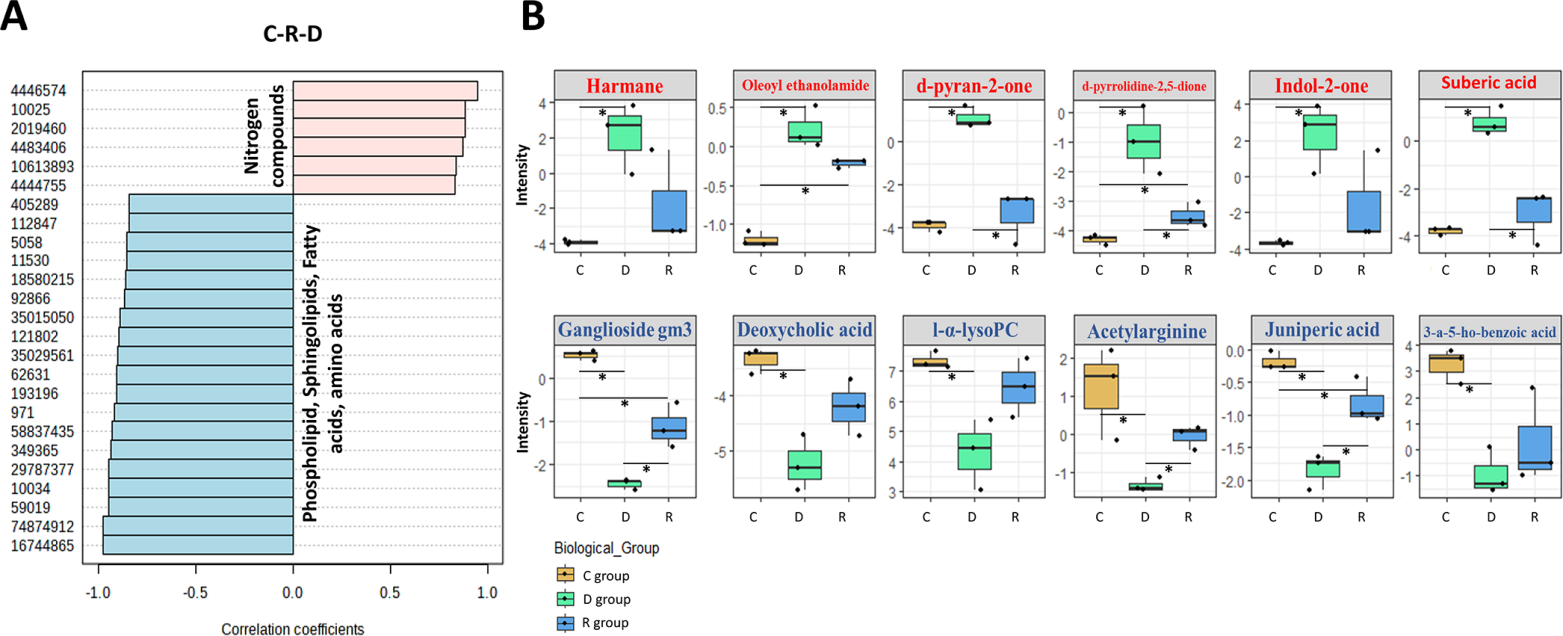
(A) PatternHunter analysis for the C, R, and D biological groups. Rose and blue bars correspond to the increased or decreased metabolites following the C-R-D pattern. Boxplots of statistically significant metabolites in at least one comparison group (D vs C, DvsR., and RvsC) with an increased or decreased pattern. (B) C, D, and R groups are represented by orange, green, and blue colors in the boxplots, respectively. *Student’s *t* test with a *p*-value < 0.05. Metabolite abbreviations: 1-(4-methyl-2-pyridyl)­pyrrolidine-2,5-dione: *d*-pyrrolidine-2,5-dione; (4*S*)-4-hydroxy-4-methyltetrahydro-2*H*-pyran-2-one: *d*-pyran-2-one; 3-amino-5-hydroxybenzoic acid: 3-a-5-ho-xybenzoic acid; l-α-lysophosphatidylcholine: l-α-lysoPC; ganglioside GM3 (d18:1/24:1­(15z)): ganglioside GM3.

Furthermore, sphingolipids, lipids, fatty acids, and amino acids decreased according to the C-R-D pattern (Figure 4B). Ganglioside GM3 and juniperic acid were found to be less abundant in infected patients, remaining altered during the recovery stage (R vs C). Acetylarginine demonstrated a negative impact, but its levels were successfully restored postinfection.

### Metabolite–Metabolite and Protein–Metabolite Interaction Networks

To uncover metabolites interacting with those significantly altered during SARS-CoV-2 infection in elderly patients (D vs C), we employed a metabolite–metabolite interaction network (Figure 5A). This approach facilitated the identification of another potentially disrupted metabolic pathway triggered by viral infection. Palmitic and oleic acids were the main nodes of the network. Notably, dopamine and adenosine emerged as nodes with the highest number of interactions. The upregulated metabolites interacted with metabolites belonging to purine and pyrimidine metabolism, biosynthesis of unsaturated fatty acids, and glycine, serine, and threonine metabolism. Conversely, downregulated metabolites demonstrated interactions with metabolites corresponding to various pathways, including arginine biosynthesis, retinol, arginine, proline, tyrosine, alanine, aspartate, glutamate, and pyruvate metabolism, glycolysis/gluconeogenesis, biosynthesis of unsaturated fatty acids, glyoxylate and dicarboxylate metabolism, and the TCA cycle.

**Figure 5:**
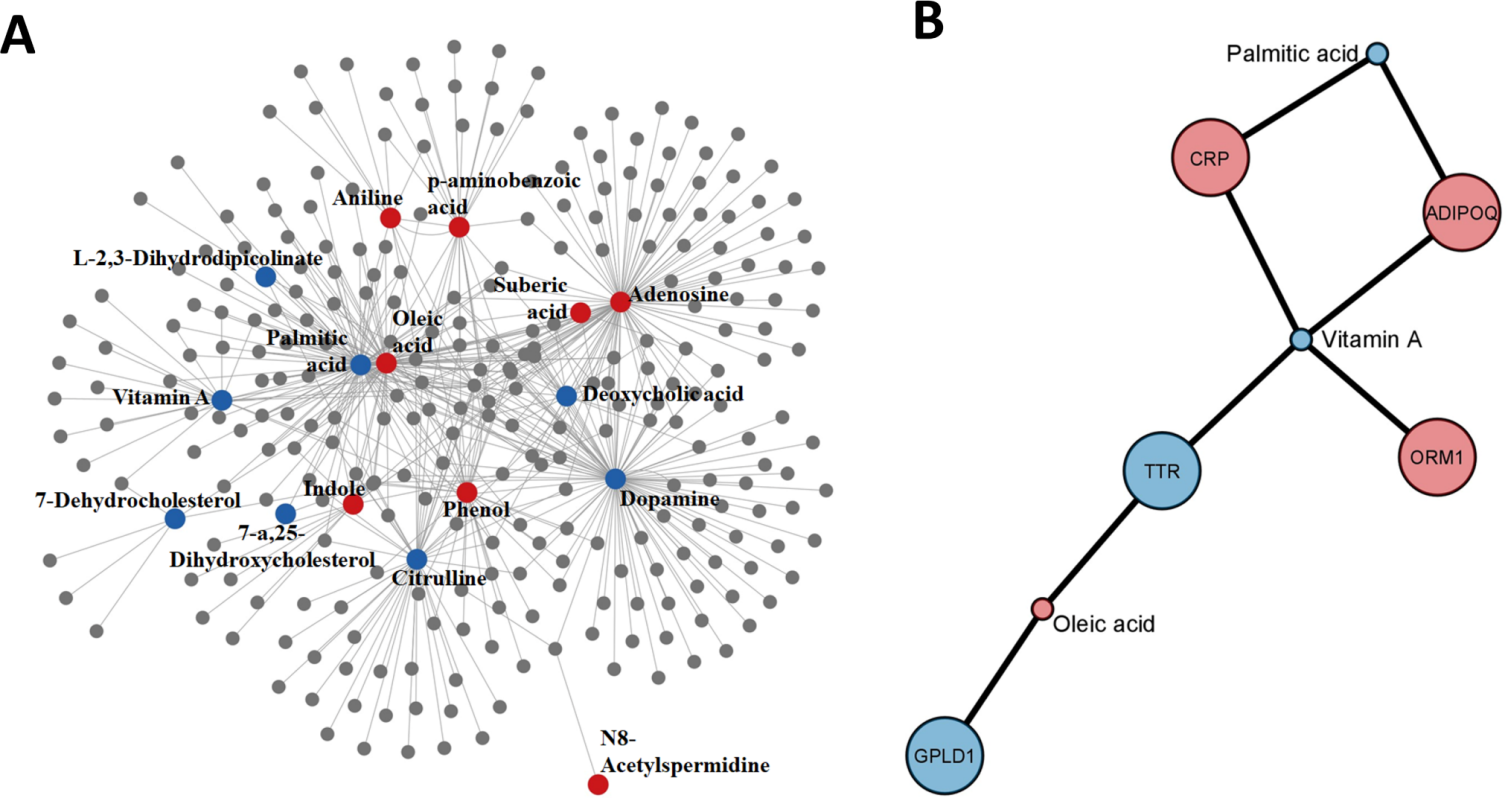
(A) Metabolite–metabolite interaction network. Red and blue circles represent the up- and down-significant metabolites in the D vs C comparison. Gray circles correspond to the interacting metabolites. (B) Protein–metabolite interaction network from the statistically significant proteins and metabolites in the D vs C comparison. Red and blue circles show up- and down-abundant proteins and metabolites, respectively.

Vitamin A, palmitic acid, and oleic acid demonstrated associations with five statistically significant proteins in the D vs C comparison (Figure 5B). The proteins CRP and ADIPOQ exhibited interactions with palmitic acid and vitamin A, with the latter also engaging with ORM1. The TTR protein showed a connection with vitamin A and oleic acid metabolites, while oleic acid also interacted with the GPLD1 protein. Intriguingly, the interacting proteins and metabolites exhibited opposite modulation.

## Discussion

In this study, we explore the molecular fingerprints observed in nonagenarian patients who experienced severe SARS-CoV-2 infection before vaccination. We also examined molecular alterations associated with recovery and mortality. To accomplish this, we compared elderly severe patients who survived (R) or did not (D) to a healthy group (C). Throughout the results section, we provide detailed descriptions of the D vs C, D vs R, and R vs C comparisons. However, the discussion will focus on D vs C and R vs C comparisons. These specific comparisons are considered more relevant as they contribute to characterizing the survival and nonsurvival stages, aligning with the main goals of our study. Furthermore, the R and C groups showed similarities in terms of their proteomic and metabolomic profiles; therefore, comparing both groups with the D group yielded similar results.

### Lipid Metabolism Impairment Associated with Severe COVID-19

The lipid metabolism in nonsurvivor nonagenarian patients (group D) was negatively impaired, as indicated by the reduced abundance of APOH, APOC1, 7-α-25-dihydroxycholesterol, and 7-dehydrocholesterol (Figures 2C and 5A). Both APOH and APOC1 abundances were confirmed through targeted proteomics analysis (Figure 1A). Cholesterol homeostasis plays a crucial role in the coronavirus life cycle, influencing viral attachment, entry, replication, maturation, and release, all mediated by cholesterol availability.^25,26^ The elevation of cholesterol and phosphatidylethanolamine (PE) in the plasma membrane enhances membrane fluidity, thereby facilitating viral entry.^27^ The diminished levels of these molecules in infected nonagenarian patients may be linked to a host response aimed at inhibiting viral entry. Similarly, the decrease in 7-α-25-dihydroxycholesterol could be associated with an antiviral response, as its precursor, 25-hydroxycholesterol, inhibits SARS-CoV-2 entry by activating the ACAT protein, leading to cholesterol depletion in the plasma membrane.^28^

A previous study analyzing risk factors for COVID-19-related mortality in people over 90 years found that HDL-cholesterol and other blood biomarkers such as 25-hydroxyvitamin D and IGF-1 were the most significant blood biomarkers of mortality.^29^ Also, other studies have reported low levels of total HDL- and LDL-cholesterol in the plasma of COVID-19 patients.^30^ These alterations, along with the increased abundance of C-reactive protein (CRP), have been linked to COVID-19 severity and mortality,^31,32^ possibly contributing to the nonsurvival outcome in the infected nonagenarian patients (Figures 5 and 2C). The CRP concentration was 128 times higher in nonsurvivor patients than in healthy controls (D vs C). The down-abundance of APOC1, APOH, and LCAT proteins has been previously identified in severe COVID-19-infected patients. Notably, alterations in the LCAT protein were linked to nonsurvivor outcomes.^33^ In this sense, the dysregulation of APOC1, APOH, and LCAT proteins in infected nonagenarian patients may serve as indicative markers of mortality, even if the statistical significance of LCAT alone did not reach a conventional threshold (*p-*value = 0.059; log2-fold change = −1). The LCAT protein is essential for cholesterol homeostasis, and its inhibition can lead to cardiovascular disease.^34^ Disrupted cholesterol regulation may be linked to a pro-atherogenic inflammatory response from SARS-CoV-2 infection,^35^ contributing to ischemic cardiovascular complications in COVID-19 patients. Therefore, monitoring LCAT enzyme levels could serve as an important marker for COVID-19 fatal outcomes.

We also found negative regulation of phosphatidylcholine (PC), PE, and sphingolipids (SM) in severe nonagenarian patients. This pattern is consistent with findings from other studies.^36–38^ The decreased levels of the ganglioside GM3 (d18:1/24:1­(15z)) metabolite in COVID-19-infected nonagenarian patients were previously correlated with disease severity.^39^ The decrease of PC, PE, and SM concentrations in infected nonagenarian patients may serve as markers indicative of nonsurvivors in elderly subjects. Alterations in host lipid metabolism with a particular focus on lipid droplets and lipid rafts were also reported for SARS-COV-2 infection.^40,41^ Phospholipids such as PC and PE, along with SM, have shown variations in the serum of severe COVID-19 patients.^38,42^ However, a consensus on the up- or down-abundance of these metabolites during COVID-19 is lacking in the literature.^37–39,42^ Conversely, higher concentrations of lipids, specifically SM, were associated with a low susceptibility to SARS-CoV-2 infection. This aligns with the behavior observed in our nonsusceptible healthy subjects (C group).^38^ Exploring the proteins and metabolites in recovered individuals could provide valuable insights into patient survival following a severe infection.

### Endogenous N-Heterocyclic Compound Increases as Anti-SARS-CoV-2

The primary observation in severe COVID-19 patients revealed elevated levels of N-heterocyclic compounds compared to healthy, nonsusceptible subjects (Figure 4B). These compounds include the pyridine, pyrrolidine, purine, pyrazole, quinoline, indole, and diazine classes. Several N-heterocyclic compounds have been demonstrated to show antiviral activity.^43,44^ This increase may indicate an antiviral response displayed by infected patients in response to the SARS-CoV-2 virus.^45^ Utilizing a molecular docking strategy, amodiaquine (quinoline class) emerged as a potential antiviral therapy by inhibiting the SARS-CoV-2 main protease.^46^ A recent study identified three synthetic pyridine derivatives exhibiting anti-SARS-CoV-2 effects through both *in silico* and *in vitro* studies.^47^ Notably, alterations in pyrimidine and purine metabolisms were previously reported in the serum of severe COVID-19 patients.^48^ Additionally, disruptions in pyrimidine metabolism may be linked to the viral replication process, with research reporting that the combination of host nucleoside biosynthesis inhibitors with antiviral nucleoside analogues produces a synergistic effect, inhibiting SARS-CoV-2 infection.^49^ Interestingly, the impairment of these N-heterocyclic compounds was fully restored after acute infection, indicating that these compounds could serve as markers for patient survival.

### Recovery Stage after SARS-CoV-2 Infection

As previously observed, the recovered severe patients exhibit minimal differences compared to healthy nonsusceptible subjects (Figures S1, 2C, and 3F). Disruptions in lipoproteins, cholesterol, and fatty acids observed during severe infection were significantly restored during the postinfection stage, as well as severity marker proteins such as CRP, SAA1, and SERPINA3. Similarly, the N-heterocyclic compounds that were impaired in severely infected patients showed no significant alterations in the recovered patients. These omics data illustrate a recovery in severely infected patients within a 3-month recovery period. Furthermore, the recovered patients appear to acquire a metabolic and proteomic profile analogous to nonsusceptible COVID-19 subjects, suggesting an enhanced defense against SARS-CoV-2. Previous reports of supercentenarians who survived COVID-19 show that these individuals displayed robust levels of IgG and neutralizing antibodies (NAbs) against SARS-CoV-2, as well as increased proteins related to an enhanced innate immune response capable of effectively neutralizing the infection.^50^

Although proteins associated with the response in the acute phase, as well as those related to the regulation of interleukin-1, tend to return in the recovered group, after three months, they do not reach the same levels as the control group (Figure 2A–C, cluster 3). Furthermore, vasoconstriction and complement activation remain altered even in the recovery phase (Figures 1E and 2A–C, cluster 3), indicating possible late effects of COVID-19 infection. The complement system may be a key driver of long COVID pathology and a potential target for diagnosis and treatment. A proteomic study that followed 113 COVID-19 patients and 39 healthy controls for up to one year showed persistent complement activation and thromboinflammation, marked by low levels of C7 complexes and high levels of C5bC6 complexes.^51^

Several metabolic alterations have been documented in recovered severe COVID-19 patients, with changes exhibiting variability across cohorts, age groups, sequelae symptoms, and the timing of sample collection in the postinfection stage. Carnitines, ketone bodies, fatty acids, LPC/PC, tryptophan, bile acids, purines, and omeprazole metabolites were found to be altered in patients with severe acute respiratory syndrome coronavirus.^52^ Plasma studies have identified an augmentation in glycerophospholipids and amino acid concentrations during the postinfection phase in severe patients, with these changes being linked to both disease survival^15^ and severity.^53^ Interestingly, four phospholipids (PC and PE) exhibited an increased abundance. Among them, two were found to decrease in deceased patients, suggesting their potential involvement in the pathogenesis of COVID-19. Although the massive impact of sphingolipids induced by SARS-CoV-2 infection has largely been restored, the crucial SM remains altered. GM3 (d18:1/24:1­(15z)), proposed as a marker for COVID-19 severity, remains consistently down-abundant in recovered patients across all comparisons. This impairment was previously documented in patients with critical infection^54^ and may be linked to the cognitive dysfunction experienced by COVID-19 patients.^55^ It is important to highlight that previous studies report high levels of sphingolipids in people who are nonsusceptible to COVID-19, which confers certain “protection.” This may cause some bias when comparing against infected patients.^38^

Further evidence of neurological detriment in recovered severe nonagenarian patients is reflected in the modulation of catecholamine metabolism, particularly dopamine. The dopamine beta-hydroxylase (DBH) protein was down-abundant in recovered patients, along with its substrate dopamine (*p-*value = 0.059), both alterations previously reported in other studies.^56,57^ Although the decrease in dopamine (D. vs C.: *p-*value = 0.036) may initially be linked to SARS-CoV-2 pathogenesis,^58^ its sustained alteration in recovered groups could indicate a different molecular disturbance, such as brain damage. Altered dopamine concentration was previously noted in the serum of COVID-19 patients.^56^ Notably, early SARS-CoV-2 variants were shown to reduce dopamine production and induce neuronal senescence in dopaminergic neurons, contributing to the process of brain aging.^59^

## Limitations

While this study provides valuable insights, it has certain limitations inherent to the characteristics of the analyzed biological groups. The sample size is limited due to the study’s focus on nonagenarians, a cohort in which unvaccinated individuals were rare but necessary to explore the specific molecular signatures of extreme aging and resilience to COVID-19. To enhance the robustness of our findings despite this constraint, we employed two complementary discovery approaches, i.e., proteomics and metabolomics, to maximize the likelihood of identifying genuine disease-related molecular alterations. The most significantly altered proteins were subsequently validated using a targeted mass-spectrometry-based assay, providing orthogonal confirmation that these results reflect true biological differences rather than statistical artifacts. Furthermore, many of the observed molecular patterns are consistent with findings reported in similar cohorts in the literature, reinforcing the validity of our discoveries. Despite these challenges, our study sheds light on unique molecular signatures in one of the oldest unvaccinated Brazilian populations infected with SARS-CoV-2 during the early pandemic.

## Conclusions

In conclusion, the nonagenarian patients that did not survive COVID-19 (Group D) revealed intriguing metabolic and proteomics alterations, particularly the pronounced impact on lipid proteins and N-heterocyclic compounds compared to healthy controls. Proteins associated with vasoconstriction and complement activation remain altered in the recovered patients (Group R), suggesting potential late effects of COVID-19. Despite a substantial recovery of sphingolipid compounds, GM3 (d18:1/24:1­(15z)) remained consistently reduced in the recovered group, presenting itself as a potential marker for COVID-19 mortality in elderly individuals. The persistent alterations of DBH protein and dopamine in both severe and recovered nonagenarian patients may be associated with long-term manifestations, such as neurological damage. In this context, the modulation of lipid metabolism in elderly patients with severe COVID-19 infection (including both survivors and nonsurvivors), as confirmed by multiomics analysis, could serve as markers for the medical management of COVID-19 patients and may potentially help reduce SARS-CoV-2 fatality rates. These findings emphasize the importance of ongoing medical follow-up during the recovery phase for individuals who have experienced severe COVID-19, particularly among older people, to address both immediate and potential long-term health implications.

## Supplementary Material

















## Data Availability

Proteomics and metabolomics raw data are available in the ProteomeXchange repository with the identifier PXD052222.

## Notes

Supplementary Figure 1: volcano plots of two-sample *t* test comparisons of (A) D vs C, (B) D vs R, and (C) R vs C from SRM results; blue and red dots represent proteins found to be downregulated or upregulated, respectively; labeled proteins were also found in the bottom-up approach (PDF)
Supplementary Table 1: clinical information and sample details (XLSX)
Supplementary Table 2: identified proteins in C, R, and D groups (XLSX)
Supplementary Table 3: ANOVA significant proteins between the C, R, and D groups (XLSX)
Supplementary Table 4: significant proteins in the Student *t* test between C, R, and D groups (XLSX)
Supplementary Table 5: metabolites identified in the C, R, and D groups (XLSX)
Supplementary Table 6: ANOVA significant metabolites between C, D, and R groups considering *p*-value <0.05 (XLSX)
Supplementary Table 7: significant metabolites in the Student *t* test applied to C, D, and R groups considering *p*-value <0.05 (XLSX)
