# Supplementary material for: Proteomics and Metabolomics Profiles of Unvaccinated Nonagenarian Patients with Severe SARS-CoV‑2 Infection
Source: J Proteome Res. 2025 Sep 25;24(11):5453–66. doi: 10.1021/acs.jproteome.5c00251 (PMC12604035; doi:10.1021/acs.jproteome.5c00251)
Supplement: Supplementary file 1 [file pr5c00251_si_001.pdf]

Supporting Information

Proteomics and metabolomics profiles of unvaccinated nonagenarian patients with severe SARS-CoV-2 infection.

Mauricio Quiñones-Vega[1,2,3#], Patricia Sosa-Acosta[1,2,3#], Jéssica de Siqueira Guedes[1,2,3], Natália Pinto de Almeida[1,2,3], Mateus V. de Castro[4], Moníze V. R. Silva[4], Luiz P. Dell'Aquila[5], Álvaro Razuk-Filho[5], Pedro B. Batista-Júnior[5], Mayana Zatz[4], Fábio César Sousa Nogueira[1,2,3*], Gilberto Barbosa Domont[1,3*].

Affiliations:
[1]Proteomics Unit, Department of Biochemistry, Institute of Chemistry, Federal University of Rio de Janeiro, Rio de Janeiro, RJ, 21941-909, Brazil; [2]Laboratory of Proteomics, LADETEC, Institute of Chemistry, Federal University of Rio de Janeiro, Rio de Janeiro, RJ, 21941-598, Brazil; [3]Precision Medicine Research Center, Institute of Biophysics Carlos Chagas Filho, Federal University of Rio de Janeiro, Rio de Janeiro, 21941-902, Brazil; [4]Human Genome and Stem Cell Research Center, University of São Paulo, São Paulo, São Paulo, 05508-000 Brazil; [5]Prevent Senior Institute, São Paulo, São Paulo, 04547-100, Brazil;

[#] Mauricio Quiñones-Vega and Patricia Sosa-Acosta contributed equally to this work.

Corresponding authors:

[*]Gilberto B. Domont: gilberto@iq.ufrj.br

[*]Fábio C. S. Nogueira: fabiocsn@iq.ufrj.br

Contents:

Supplementary figure 1. Volcano plots of two-sample t-test comparison of A) D vs C B) D vs R and C) R vs C from SRM results. Blue and red dots represent proteins found down or up-regulated respectively. Labelled proteins were also found in bottom-up approach.

Supplementary Table 1. Clinical information and sample details

Supplementary Table 2. Identified Proteins in C, R and D groups

Supplementary Table 3. ANOVA significant proteins between the C, R and D groups

Supplementary Table 4. Significant proteins in Student´s t-test between C, R and D groups

Supplementary Table 5. Metabolites identified in the C, R, and D groups.

Supplementary Table 6. ANOVA significant metabolites between C, D, and R groups considering p-value < 0.05.

Supplementary Table 7. Significant metabolites in Stundent´s t-test applied to C, D, and R groups considering p-value < 0.05.

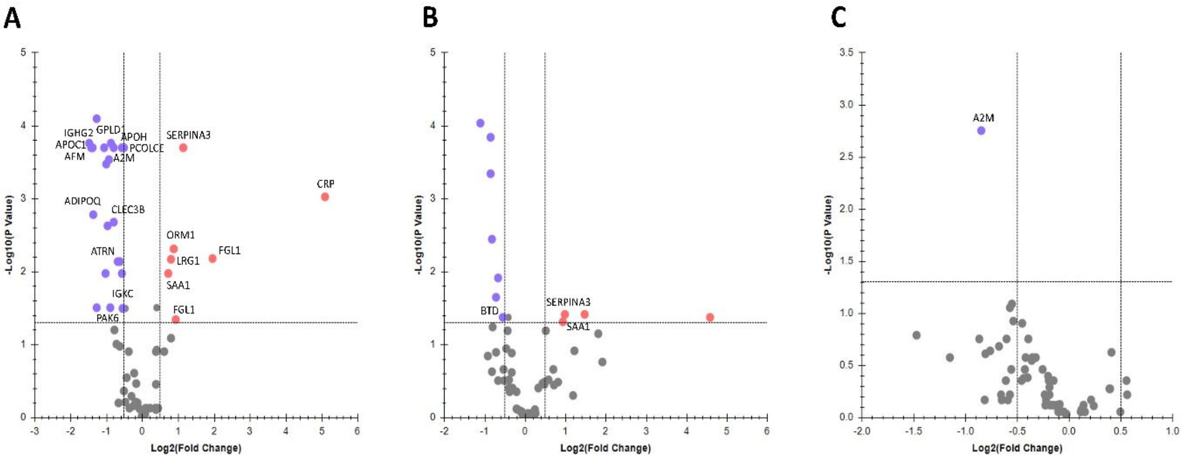

Supplementary Figure 1. Volcano plots of two-sample t-test comparison of A) D vs C B) D vs R and C) R vs C from SRM results. Blue and red dots represent proteins found down or up-regulated respectively. Labelled proteins were also found in bottom-up approach.
